# The Pro-tumorigenic IL-33 Involved in Antitumor Immunity: A Yin and Yang Cytokine

**DOI:** 10.3389/fimmu.2018.02506

**Published:** 2018-10-26

**Authors:** Jean-Jacques Fournié, Mary Poupot

**Affiliations:** INSERM UMR 1037 Centre de Recherche en Cancérologie de Toulouse (CRCT), ERL 5294 CNRS, Université Toulouse III Paul Sabatier, Laboratoire d'excellence Toucan, Toulouse, France

**Keywords:** interleukin-33, immunity, cancer, immunosuppression, microenvironment

## Abstract

Interleukin-33 (IL-33), considered as an alarmin released upon tissue stress or damage, is a member of the IL-1 family and binds the ST2 receptor. First described as a potent initiator of type 2 immune responses through the activation of T helper 2 (T_H_2) cells and mast cells, IL-33 is now also known as an effective stimulator of T_H_1 immune cells, natural killer (NK) cells, iNKT cells, and CD8 T lymphocytes. Moreover, IL-33 was shown to play an important role in several cancers due to its pro and anti-tumorigenic functions. Currently, IL-33 is a possible inducer and prognostic marker of cancer development with a direct effect on tumor cells promoting tumorigenesis, proliferation, survival, and metastasis. IL-33 also promotes tumor growth and metastasis by remodeling the tumor microenvironment (TME) and inducing angiogenesis. IL-33 favors tumor progression through the immune system by inducing M2 macrophage polarization and tumor infiltration, and upon activation of immunosuppressive cells such as myeloid-derived suppressor cells (MDSC) or regulatory T cells. The anti-tumor functions of IL-33 also depend on infiltrated immune cells displaying T_H_1 responses. This review therefore summarizes the dual role of this cytokine in cancer and suggests that new proposals for IL-33-based cancer immunotherapies should be considered with caution.

## Introduction

Cancer development depends on hallmarks such as self-sufficient proliferation, escape to anti-apoptotic signals, resistance to apoptosis, immune evasion, infinite replication, nurture of vascularization, and ability for invasion and metastasis (1). However, these hallmarks do not only concern the cancer cell but also the tumor microenvironment (TME) which is essential for tumorigenesis. The TME consists of fibroblasts, endothelial cells, immune cells, pericytes, and smooth muscle cells which are recruited by cancer cells as non-malignant cells but then modified to take part in tumor development (2–4). Besides cellular components, acellular components such as matrix, chemokines, and cytokines are also essential for tumor development (4). Cytokines as central mediators, favor the interaction between cells in the inflammatory tumor microenvironment (5). Amongst these cytokines, Interleukin-33 (IL-33), a member of the IL-1 superfamily of cytokines (6), is well-known now to have an important role in innate and adaptive immunity through its contribution to tissue homeostasis and responses to stress such as tumor development. IL-33 is constitutively expressed at high levels in the nucleus of human and mouse tissue lining and in various cell types including vascular endothelium (7), endothelial cells of endothelial venules (HEVs) (8, 9) and epithelial cells in barrier tissues that are exposed to the environment such as bronchial epithelial cells (10), keratinocytes, epithelial cells of the stomach, and salivary glands (7). Fibroblastic reticular cells (FRCs) in lymphoid tissues and cells of the central nervous system represent a major source of IL-33 (7, 11). IL-33 was first described in HEV as an intracellular nuclear factor with transcriptional regulatory properties (8). It was then shown that IL-33 binds a heterodimer formed by the specific ST2 receptor and a co-receptor, the IL-1 receptor accessory protein (6, 12). To exert its cytokine activity and alert the immune system, IL-33 is not secreted extracellularly like a conventional cytokine but after cell injury following cell stress or damage (11, 13–16). Full-length IL-33 is thus considered as an alarmin produced as a result of an injury to the central nervous system (15), a mechanical stress (17, 18), necroptosis (19) but also in pathological wound repair and fibrosis (20–22). The IL-33/ST2 axis is also associated with many inflammatory diseases such as asthma (23–25), rheumatoid arthritis, psoriatic arthritis or osteoarthritis (26), pulmonary fibrosis (27) or dermatitis and allergic contact dermatitis (28, 29). Many publications have summarized the important role of IL-33 in these diverse inflammatory diseases (30–34). IL-33/ST2 signaling is transduced by MyD88 and the kinase-4 associated to the ST2 receptor, which is a downstream adaptor protein, shared with other IL-1 family members and Toll-like receptors (35). Moreover, the soluble form of ST2 (sST2) produced from 3′-UTR promoter or splice variants mRNA, can be a decoy receptor for IL-33 (36–38). IL-33 was first described as a potent initiator of type 2 immune responses through the activation of many cell types, including the T_H_2 subset of helper cells, type 2 innate lymphoid cells (ILC2s), mast cells, basophils, eosinophils, and myeloid cells such as myeloid-derived antigen-presenting cells including macrophages and dendritic cells (DCs) (6, 35, 39–45). Furthermore, IL-33-exposed DCs or mast cells also selectively support FOXP3^+^ regulatory T cell (T_reg_ cells) expansion through IL-33-induced secretion of IL-2 (40, 41) and favor T_H_17 cell differentiation through IL-1β and IL-6 secretion (46). IL-33 was detected in the serum of patients with T_H_1/T_H_17 mediated diseases (47, 48). However, besides this pro-inflammatory function of IL-33, its protective role in atherosclerosis, obesity, type2 diabetes and cardiac remodeling also holds an important place (16, 49–51). Moreover, IL-33 can also activate type 1 immune responses via TNF-α and IFN-γ expression by CD8 T lymphocytes, natural killer (NK) cells or iNKT cells. The latter can be stimulated by IL-33 upon its ligation to their cell surface ST2 receptors (13, 52–55). Finally, several studies have shown an important involvement of IL-33 in several types of cancer with pro or anti-tumorigenic functions depending on the immune status of the tumor. The goal of this review is to summarize the hallmarks of IL-33 in cancer, both in terms of its pro-tumorigenic function targeting resident T_H_2 immune cells of the TME, and as a tumor suppressor molecule activating the competent T_H_1 immune cells.

These properties therefore position IL-33 as a possible inducer and prognostic marker of cancer development, as reviewed here.

## IL-33 as a marker for good or poor prognosis

IL-33 has been shown to be a promising biomarker in several types of cancer for tumor detection and as a predictor of prognosis and therapeutic response. Recently, IL-33 was shown to be correlated with a bad prognosis in several types of cancer, although in some cases IL-33 behaves as a tumor suppressor by inducing an immune response. In terms of bad prognosis, high levels of IL-33 were detected in the serum and tumors of patients with glioma (56), gastric cancer (57), hepatocellular carcinoma (58), uterine leiomyoma (59), lung cancer (60), colorectal cancer (61), head and neck squamous cell carcinoma (62), and breast cancer (63), when compared to corresponding healthy tissues. The Cancer Genome Atlas Pan-Cancer analysis project showed and declared that the level of IL-33 expression is altered in only 3% of ~580 tumors and that the most common genetic alteration is the deletion of the IL-33 gene (64).

Lu and collaborators detected “significantly higher IL-33 expression in glioma tissues than in normal brain tissues through immune-histochemical (IHC) analysis” (56). High IL-33 expression in glioma was correlated with shorter overall survival (OS) and progression-free survival (PFS) (56). In women, IL-33 highly promotes epithelial cell proliferation and tumorigenesis in breast cancer, since IL-33 increases Cancer Osaka Thyroid (COT) phosphorylation via ST2-COT interaction in normal epithelial and breast cancer cells. This induces the activation of MEK-ERK, JNK-cJun, and STAT3 signaling pathways, both leading to cell proliferation (65). The expression levels of IL-33 and ST2 proteins were also positively correlated with the expression of Ki-67 in epithelial ovarian cancer tumors and at the metastatic site, and negatively correlated with the patient survival time (66). High expression of IL-33, assessed by IHC staining, was associated with advanced stage clear-cell renal carcinoma and abnormally high amounts of serum IL-33 was detected in patients with hepatocellular carcinoma or gastric cancer. Hence, IL-33 is correlated with a bad prognosis in these types of cancer (57, 58, 67). sST2 was also described as a negative prognostic marker when its serum concentration was associated with OS of patients with hepatocellular carcinoma (68). Nevertheless, this soluble IL-33 receptor can also be associated with a good prognosis in colorectal cancer, as the trapping of soluble IL-33 in the TME inhibits cancer growth and metastases (69). Moreover, the level of IL-33 protein has been inversely correlated with tumor grade and size in patients with pulmonary adenocarcinoma, showing an association of low IL-33 expression level with a poor prognosis (70–72). Likewise, a genome-wide association study unveiled a correlation between high IL-33 expression and a good prognosis in patients with osteosarcoma (73). However, if IL-33 can have a pro-tumor effect by directly targeting cancer cells, the tumor suppressor functions displayed by IL-33 are indirectly promulgated by immune surveillance as we will show hereafter.

## IL-33 as a pro-tumorigenic cytokine through actions on cancer cells and TME

As described above, IL-33 is considered as a prognostic biomarker when expressed in tumors. This intratumoral IL-33 is expressed by cancer cells as well as by other cell components of the TME. For instance, in patients with head and neck squamous cell carcinoma and oral squamous cell carcinoma, intratumoral IL-33 has been shown to be expressed in cancer-associated fibroblasts (CAF) (62, 74). This infiltrating IL-33 has a direct pro-tumorigenic effect on cancer cells and indirect effects on cellular components of the TME.

In the first case, Wang and collaborators showed that the IL-33/ST2 pathway up-regulated membrane glucose transporter 1 in non-small-cell lung cancer cells, enhancing their glucose uptake and glycolysis, thus favoring *in vitro* outgrowth of human lung cancer and its metastasis in a mouse model (60). By *in vitro* and *in vivo* experiments, IL-33 was also shown to be able to promote growth, invasion and migration of gastric cancer and colorectal cancer cells due to the autocrine secretion of several metalloproteases (MMP3, MMP9, MMP2), IL-6 and CXCR4 via the ST2-ERK1/2 pathway (61, 75). Moreover, IL-33 directly targets colon cancer cells and breast cancer cells via JNK-cJun activation, which promotes cell proliferation and therefore tumor growth (65, 76).

The impact of IL-33 on the TME encompasses angiogenesis, matrix remodeling and cytokine/growth factor production by non-epithelial cell components. The IL-33/ST2 signaling pathway, favoring pro-angiogenic VEGF expression in tumor cells and reducing tumor necrosis, is highly involved in mammary tumor growth (77). Concerning matrix modeling, human subepithelial myofibroblasts stimulated *in vitro* with IL-33 induced the expression of extracellular matrix components and growth factors associated with intestinal tumor progression (78). IL-33-stimulated cancer cells produce cytokines, and TME infiltrating immune cells are also involved in the expression of IL-6 in response to IL-33/ST2 signaling. Likewise, IL-33 stimulates the secretion of cytokines and growth factors in bone marrow myeloid and non-hematopoietic cells, resulting in myeloproliferation of neoplasms (79, 80). Indeed, suppression of IL-33 or high expression of sST2 suppresses IL-33-induced angiogenesis, T_H_2 responses, macrophage infiltration and M2 macrophage polarization. This negatively regulates tumor growth and metastatic spread of colorectal cancer, for instance through the modification of the TME (69). IL-33 in the TME recruits macrophages and stimulates their production of PGE_2_, and in turn, macrophage-derived PGE_2_ stimulates colon tumor development (76). The recruitment of macrophages in the TME might account for the stimulation of CCL2 expression by IL-33-stimulated cancer cells that express ST2, such as human colon cancer cells (81). After recruitment, macrophages are directly induced by IL-33 to be polarized in M2 tumor associated macrophages (TAM) in the TME. Such TAMs are then able to produce IL-10, VEGF, IL-6, and MMP9 which promote proliferation and invasiveness of cancer cells (82–84). Yang and collaborators showed that TAM are recruited by IL-33 in the TME, and IL-33-stimulated TAM can increase intravasation of tumor cells into the circulation at the early stages of metastasis (85). Even in the brain, IL-33 in the TME induces growth of glioma cells and facilitates microglia/macrophage infiltration (86). IL-33-stimulated macrophages are also activated to produce G-CSF, which in turn, boost myeloid-derived suppressor cells (MDSC) from the pro-tumoral TME (87). Indeed, MDSC contribute to tumor-mediated immune escape by suppressing antitumor immune responses. IL-33 released in tumor tissues in breast and colorectal cancer mouse models and in breast cancer patients, has been shown to facilitate MDSC expansion, recruitment and survival in the TME. This role could be due to the induction of an autocrine secretion of GM-CSF (88–90). Interestingly, another study showed that IL-33 does not affect the number of MDSC but can significantly reduce the differentiation of lineage-negative bone marrow progenitor cells into granulocytic MDSC in tumor-bearing mice. Moreover in the same study, IL-33-treated MDSC were shown to be less immunosuppressive, with a reduced capacity to inhibit T cell proliferation and IFN-γ production, production of reactive oxygen species and their capacity to induce T_reg_ differentiation and expansion (91). IL-33 has a direct effect on T_reg_ cells expressing surface ST2. Indeed, these lymphocytes are constitutively abundant in the intestine and able to prevent dysregulated inflammatory responses to self and environmental stimuli. IL-33 is constitutively expressed in epithelial cells at barrier sites. High levels of IL-33 were also observed in inflamed lesions of patients with inflammatory bowel disease, supporting its role in disease pathogenesis (92, 93). In inflammatory conditions, IL-33 signaling in T_reg_ cells enhances transforming growth factor (TGF)-β1-mediated differentiation. Alternatively, IL-33 may provide a signal necessary for inducing their accumulation and maintenance in inflamed tissues (94). Local accumulation of T_reg_ cells has been described in intestinal tumors preventing tumor clearance in mouse models and in patients. This role may be associated with a reduction of E-cadherin expression, increased β-catenin signaling and IL-33 production by malignant and injured epithelial cells (95). In tumors with low levels of infiltrating T_reg_ cells, administration of IL-33 accelerates tumor growth and occurrence of liver and lung metastasis in breast cancer mouse models, and these models display an intratumoral accumulation of MDSC and T_reg_ cells, as compared to untreated mice (90). Moreover, IL-33 blockade, in addition to abrogating the polarization of TAM, reduces the accumulation of T_reg_ cells in lung tumors of human lung preclinical mouse models (82). However, as inflammation contributes to tumorigenesis, the accumulation of T_reg_ in inflammatory zones must contain inflammation and therefore tumorigenesis. T_reg_ may promote or inhibit tumor development depending on the context, revealing the complex relationship between inflammation, and cancer development. Furthermore, mast cells which also express ST2 receptors and respond to cell injury via IL-33 released from necrotic cells, can secrete leukotrienes and cytokines to initiate pro-inflammatory responses (96). In a colorectal cancer mouse model, IL-33 deficiency reduced mast cell accumulation in tumors. This deficiency further inhibited the expression of mast cell-derived proteases and cytokines that promote polyposis (78, 96–98). Generally, mast cells accumulate in inflamed gut and in colorectal tumors, and their presence is correlated with a poor prognosis and low overall survival (99, 100). In skin cancers, dermal mast cells are able to respond to UVB-induced IL-33 by releasing IL-10 to protect skin homeostasis after excessive UVB exposure. However, IL-10 may contribute to skin cancer development, as IL-10-deficient mice do not develop skin tumors upon UVB exposure (101, 102).

## Direct or indirect effects of IL-33 as a tumor suppressor

Alongside its pro-tumorigenic role, IL-33 can also behave as a tumor suppressor. Only one study has shown a direct anti-tumor effect of this cytokine with the *in vitro* inhibition of proliferation and induction of apoptosis of MIA PaCa-2, a pancreatic cancer cell line (103). However, its anti-tumor functions were largely associated with the activation of immune effector cells able to lead to tumor clearance. All immune cells express the ST2 receptor and are able to respond to IL-33 stimulation. IL-33 has a significant role in cancer immune-surveillance in primary prostate and lung tumors, which can be lost during the metastatic transition inducing immune escape. The correlation between IL-33 and HLA expression in human tumors using RNA-sequencing data of resected prostate tumors was recently shown. The down-regulation of IL-33 during the metastatic process ultimately decreases the functionality of HLA-I and reduces immune-surveillance favoring tumor development (104). In a multivariable analysis, the infiltration of human hepatocellular carcinomas (HCC) by cells expressing IL-33 and by CD8^+^ T cells was associated with prolonged patient survival. These results led to propose an HCC immune score identifying high- vs. low-risk patients with different gene expression profiles (105). Injection of IL-33 into established murine melanoma or acute myeloid leukemia models inhibits tumor growth in a CD8^+^ T cell-dependent manner prolonging the survival of mice. In the first model, the reduction of tumor growth delay was correlated with intratumoral accumulation of CD8^+^ T cells, and a decrease in the number of immunosuppressive myeloid cells (106). In the second model, the anti-leukemia activity was associated with increased expansion and IFN-γ production of leukemia-reactive CD8^+^ T cells (107). Moreover, the correlation between decreased IFN-γ secretion and colon cancer aggressiveness, suggests that IL-33 signaling defects may impair the generation of IFN-γ-mediated immunity (108). In soft tissue sarcoma, higher transcriptional levels of IL-33 were also associated with a good prognosis. The expression of IL-33 has also been negatively correlated with the expression of chemokines, such as TGF-β, recruiting T_reg_ and MDSC, and positively correlated with the expression of chemokines that recruit CD8^+^ T cells which promote anti-tumor immune responses especially through INF-γ production (109).

It has been shown that IFN-γ-producing cells present in tumors associated with an IL-33 antitumor effect, were CD8^+^ T cells and NK cells. Indeed, IL-33 expression in several cancers affects the number of CD8^+^ T cells and NK cells in tumor tissues and the production of IFN-γ/TNF-α, thereby favoring tumor eradication through tumor cell cytolysis (110, 111). This was also shown with the reduction of tumor metastasis in B16 melanoma and Lewis lung carcinoma metastatic models thanks to the transgenic expression of IL-33. In these transgenic mice models, tumor infiltration and CD8^+^ T lymphocyte and NK cell cytotoxicity was significantly increased compared to non-transgenic mice. Moreover, treatment with recombinant IL-33 increased CD8^+^ T lymphocyte and NK cell cytotoxicity *in vitro* (112). CD8^+^ T cells are also indirectly stimulated by IL-33 through DC. DC maturation is promoted by IL-33 which increases their cross presentation ability particularly during the anti-leukemia or anti-melanoma immune response (107, 113). As mentioned in the introduction, IL-33-activated DC are also able to promote the differentiation of T_H_17 cells which play an important role in cancer development. T_H_17 are T helper lymphocytes secreting IL-17 and other inflammatory cytokines, but can also display immunosuppressive activities, therefore mediating context dependent pro- or anti-tumor responses (114, 115). However, there are no published studies mentioning a direct relationship between T_H_17 cells and IL-33 in cancers. T_H_17 cells expressing the ST2 receptor were found to accumulate in the small intestine in bowel diseases where intestinal epithelial cells are the providing source of IL-33, we can therefore stipulate that these cells could play a role in digestive cancers. T_H_17 cells could have an anti-tumor function with the production of pro-inflammatory cytokines (116) or a pro-tumor role when they can acquire a regulatory phenotype with immunosuppressive properties upon IL-33 activation (117). Furthermore, DC can also drive T_H_9 cell dependent anti-tumor responses through the expression of Ox40L when activated by IL-33 and stimulated by dectin-1 signaling (118–120).

Finally, the ILC2s which support type 2 immune responses by producing IL-5 and IL-13 in response to IL-33 could also have an antitumor function. Indeed, their tissue-repair function can induce cholangiocarcinoma and liver metastasis (121). ILC2s can also be mobilized from the lung and other tissues thanks to IL-33, to penetrate tumors, mediate immune-surveillance with DC, and promote adaptive cytolytic T cell responses and attraction (122, 123).

## Concluding remarks

**Figure 1:**
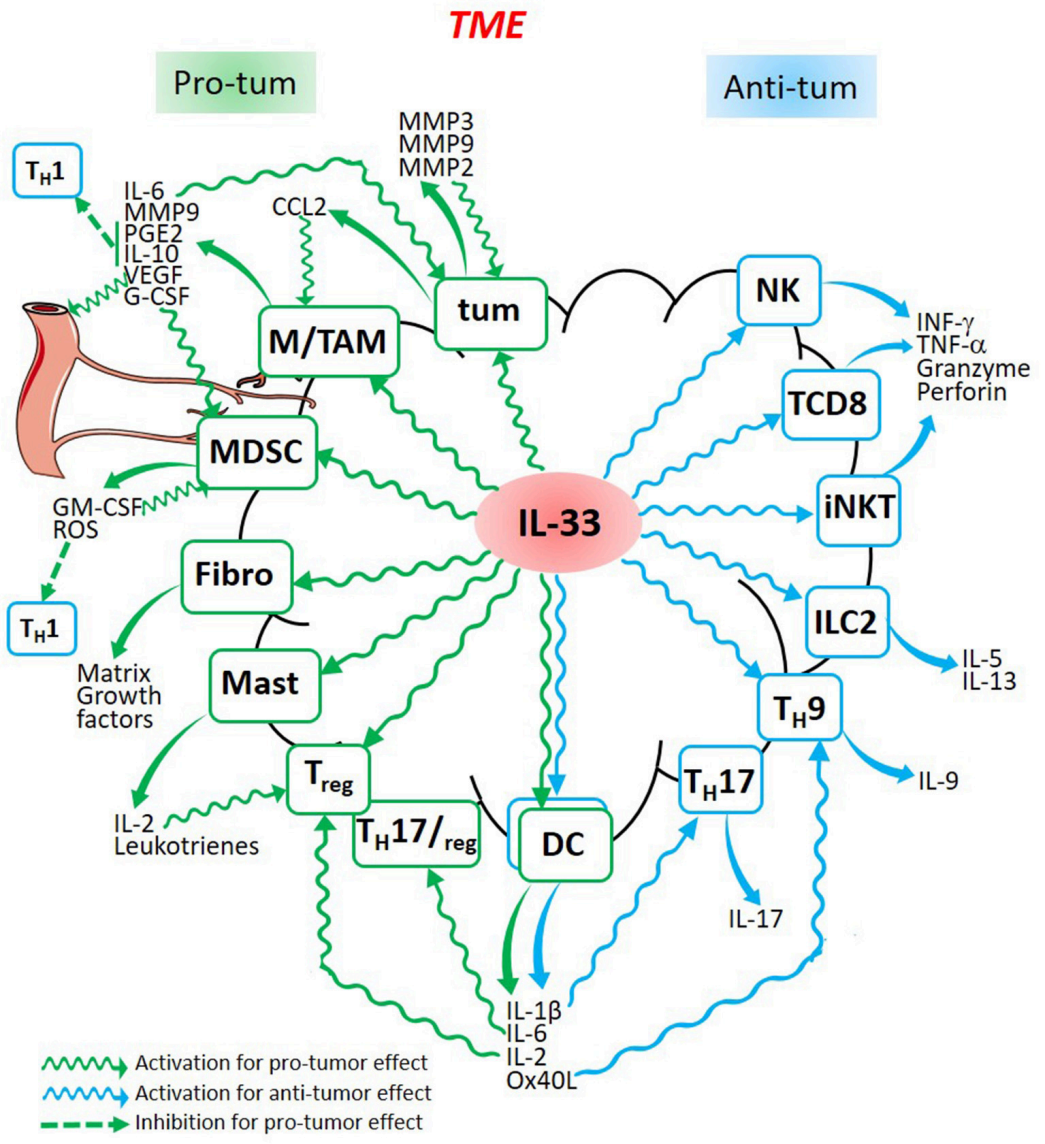
Dual role of Il-33 in cancer. IL-33 released in the TME is able to stimulate cancer cells (tum), fibroblasts (Fibro), and different immune cells (Macrophages, TAM, MDSC, mast cells, T_reg_, Dendritic Cells, T_H_17, T_H_9, ILC2, iNKT, CD8^+^ T cells, and Natural Killer cells) which are activated to produce molecules involved in pro-tumor (green) or anti-tumor (blue) processes leading to the development or to the regression of the tumor. Some cytokines produced by pro-tumor cells such as MDSC or TAM, are also able to produce cytokines which inhibit anti-tumor cells such as all the T_H_1 cells.

IL-33 therefore appears as a pro-tumorigenic cytokine that can also limit tumor growth through the activation of antitumor immunity. These opposing roles in tumorigenesis, as shown in this review, greatly depend on the IL-33/ST2 signaling in different immune cells. IL-33 is able to promote inflammatory events which contribute to tumorigenesis whilst activating anti-tumor immune responses. The different events promoted by IL-33 activation of various immune cells which can be found in the TME are summarized in the Figure 1. Depending on the tumor context, IL-33 produced in the TME can activate diverse immune cells which are able to promote a pro-tumor effect such as TAM, MDSC, fibroblasts, mast cells, T_reg_ and DC, or to prevent tumor development such as NK cells, CD8^+^ T cells, iNKT, ILC2, T_H_9, and T_H_17. All these cell types produce specific cytokines, chemokines, and other molecules. These conclusions are supported by Wasmer and Krebs' review who demonstrated the multiple functions of IL-33 in different cancer types (124). As many cytokines with immunomodulatory properties, IL-33 has been considered for anticancer immunotherapies. However, knowing its dual role, therapeutic manipulation of this cytokine should be considered with caution. The majority of the studies mentioned propose cancer immunotherapy strategies based on exogenous IL-33 administration. These IL-33 adjuvanted vaccines aim at activating the immune cells involved in the immune response (106, 107, 125–128). IL-33 could also indirectly activate effector T cells. For instance, a replicating viral vector system used in cancer immunotherapy which delivers tumor-associated antigens to DC for efficient cytotoxic T cells priming, depends on IL-33 signaling (129). IL-33 could likewise increase T cell activation to promote graft- vs.-leukemia (GVL) reactions while decreasing fatal graft-vs.-host- disease (GVHD) (130). Possibly however, ST2 blockade might preserve GVL activity by blocking T_reg_ controlling GVHD (131). Indeed, considering the immunosuppressive pro-tumorigenic role of IL-33, others have proposed to block IL-33 as a novel anticancer strategy (62, 65, 69, 76, 82, 88). In the future, IL-33 targeting in cancer immunotherapies should be considered with caution, especially taking into account the intricate dual role of this cytokine in cancer as shown in this review.

## Author contributions

All authors listed have made a substantial, direct and intellectual contribution to the work, and approved it for publication.

### Conflict of interest statement

The authors declare that the research was conducted in the absence of any commercial or financial relationships that could be construed as a potential conflict of interest.
